# Nicotinic Acid Restriction Potentiates NAMPT Inhibition in Acute Myeloid Leukemia Xenograft Models

**DOI:** 10.1158/2767-9764.CRC-26-0274

**Published:** 2026-10-28

**Authors:** Kyoji Tsurumi, Miyuki Nomura, Mai Ohuchi, Ryoma Kizu, Naohiko Kikuchi, Taku Sato, Mami Morita, Yoji Yamashita, Kayoko Hayashi, Tomoyoshi Soga, Yuki Sugiura, Tatsuro Fukuhara, Nobuhiro Tanuma

**Affiliations:** 1 Division of Cancer Chemotherapy, Miyagi Cancer Center Research Institute, Natori, Japan.; 2 Department of Biochemical Oncology, Tohoku University Graduate School of Medicine, Sendai, Japan.; 3 Department of Respiratory Medicine, Miyagi Cancer Center Hospital, Natori, Japan.; 4 Multi Omics Platform, Center for Cancer Immunotherapy and Immunobiology, Graduate School of Medicine, Kyoto University, Kyoto, Japan.; 5 Human Biology-Microbiome-Quantum Research Center, Keio University, Tokyo, Japan.

## Abstract

Nicotinamide phosphoribosyltransferase (NAMPT), a key enzyme in NAD biosynthesis, is a potential therapeutic target in cancer. Acute myeloid leukemia (AML) is among cancer types exhibiting high NAMPT dependence, and elevated NAMPT activity is implicated in venetoclax resistance. Thus, pharmacologic NAMPT inhibitors (NAMPTi) have been developed as a promising therapeutic approach in some cancers. However, our current knowledge of *in vivo* factors that determine NAMPTi therapeutic efficacy is incomplete. In this study, using mouse models we found that NAMPTi efficacy against AML is limited by dietary nicotinic acid (NA). Specifically, NA and NA riboside (NAR), an NA-derived metabolite, promoted NAD biosynthesis independent of NAMPT and rescued AML cells from NAMPTi-mediated toxicity *in vitro*. Importantly, in AML xenograft models, dietary NA restriction, which selectively reduces circulating NAR levels, enhanced antitumor efficacy of NAMPTis and prolonged animal survival. Although the combination treatment caused transient hematologic toxicity, peripheral blood cell counts largely recovered after treatment. Our findings highlight systemic NAD precursor metabolism, including NAR, as a determinant of responses to NAMPT-targeted therapies. We conclude that NA restriction may improve the therapeutic efficacy of NAMPTis in AML.

**Significance:**

NAMPT is a potential therapeutic target in AML, but factors governing treatment response remain undefined. Building on findings that dietary NA maintains circulating NAR levels, we demonstrate that this pathway provides a critical escape mechanism from NAMPT inhibition. We show that NA restriction sensitizes AML to NAMPTis, significantly improving survival *in vivo*, identifying systemic NAD precursor metabolism as a key determinant of therapeutic efficacy.

## Introduction

NAD (NAD^+^ and NADH) is a metabolic cofactor essential for cellular metabolism, serving as a redox carrier to sustain energy production and biomass synthesis required for cell proliferation (1–3). In addition to this cofactor role, NAD also serves as a precursor for NADPH synthesis and substrate for NAD-consuming enzymes, including PARPs and sirtuins (1–3). These nonredox, NAD-consuming processes reduce the NAD pool, necessitating continuous NAD biosynthesis to maintain essential cellular functions, including proliferation. In vertebrates, NAD can be synthesized from tryptophan (Trp) or niacin-class vitamins in cells capable of metabolizing those factors (4). However, in most cells, including cancer cells, the salvage pathway from nicotinamide (Nam) is considered the predominant route for NAD biosynthesis, with Nam phosphoribosyltransferase (NAMPT) acting as the rate-limiting enzyme (4–11). Accordingly, NAMPT has been considered a promising therapeutic target against certain cancers (5–12).

Recent studies show that sensitivity to NAMPT inhibition varies across cancers. However, these observations are largely derived from *in vitro* studies, and less is known about factors that determine vulnerability to NAMPT inhibition *in vivo*. In fact, standard culture media lack several NAD precursors, including nicotinic acid (NA), NA riboside (NAR), and Nam riboside (NR), all of which are present in physiologic environments (13–15), emphasizing the need for *in vivo* analysis. We recently reported that NAD synthesis from circulating NAR can compensate for NAMPT inhibition in *in vivo* models of neuroendocrine carcinoma (NEC; ref. 16). Importantly, circulating NAR is generated from dietary NA through host metabolism (16, 17). Notably, our study also indicated that restriction of dietary NA specifically and significantly reduces circulating NAR levels with minimal effects on other NAD precursors (16). Thus, combining NAMPT inhibition with NA restriction is synthetic lethal to NEC (18).

Acute myeloid leukemia (AML) is a cancer type exhibiting high NAMPT dependence (16, 19, 20). Subedi and colleagues (19) reported that AML leukemic stem cells (LSC) show greater NAMPT dependence than non-LSC populations or normal hematopoietic stem cells (HSC). Jones and colleagues (20) reported that elevated NAMPT activity in AML mediated venetoclax resistance. NAMPT inhibition has been shown to reduce NAD levels and induce apoptosis in AML LSCs (19, 20). Consistent with observations in primary AML cells, AML cell lines also exhibit high NAMPT dependence *in vitro* (16). Nevertheless, whether AML cells compensate for NAMPT inhibition through alternative NAD biosynthetic pathways has not been systematically investigated.

Here, we demonstrate that AML cells can utilize NAR to sustain NAD levels following NAMPT inhibition *in vitro* and that dietary NA restriction significantly potentiates the efficacy of pharmacologic NAMPT inhibition in AML xenograft models. We also report the effects of NAMPT inhibition, with or without NA restriction, on normal mouse hematopoiesis.

## Materials and Methods

### Reagents

Nam, NA, NR, β-Nam mononucleotide (NMN), D-glyceraldehyde 3-phosphate (G3P), and D-(−)-3-phosphoglyceric acid (3-PG) were purchased from Merck. NAR was purchased from Toronto Research Chemicals or BLD Pharm. FK866 was obtained from Cayman Chemical. GNE-617 was purchased from MedChemExpress. Allophycocyanin (APC)-conjugated anti–human CD45 (hCD45; BioLegend, cat. #304037, RRID: AB_2562049) and FITC-conjugated anti–mouse CD45 (mCD45; BioLegend, cat. #103107, RRID: AB_312972) were used for flow cytometry analysis.

### Human cell lines

Human AML cell lines U937 [Japanese Collection of Research Bioresources (JCRB) Cell Bank, JCRB9021, RRID: CVCL_0007], MOLM-14 (JCRB Cell Bank, JCRB1812, RRID: CVCL_7916), NOMO-1 (JCRB Cell Bank, IFO50474, RRID: CVCL_1609), and EoL-1 (RIKEN BioResource Research Center, RCB0641, RRID: CVCL_0258) were maintained in RPMI-1640 medium supplemented with 10% FCS. All lines were verified as *Mycoplasma* free using a MycoAlert kit (Lonza). Cell line authentication tests were not performed. All cells were cultured under 5% CO_2_ at 37°C.

EoL-1 cells were obtained in 2021, whereas NOMO-1, U937, and MOLM-14 cells were obtained in 2023. The most recent *Mycoplasma* testing was performed on September 14, 2026. The number of passages between thawing and use in the described experiments was within 10 to 12 passages.

### Lentiviral gene expression

Luciferase-expressing U937 and EoL-1 lines were generated using a lentiviral transduction system. Lentiviral particles were produced in HEK293T cells (RIKEN BioResource Research Center, RCB2202, RRID: CVCL_0063) by cotransfection of pLenti CMV Puro LUC (w168-1; Addgene plasmid #17477, RRID: Addgene_17477), psPAX2 (Addgene plasmid #12260, RRID: Addgene_12260), and pMD2.G (Addgene plasmid #12259, RRID: Addgene_12259) using FuGENE HD transfection reagent (Promega), as described previously (21). Viral supernatants were collected 48 hours after transfection and used to transduce U937 and EoL-1 cells. Transduced cells were selected with 1 μg/mL puromycin to establish stable luciferase-expressing lines. All cell lines were cultured in RPMI-1640 supplemented with 10% fetal bovine serum at 37°C in a humidified incubator with 5% CO_2_.

### Analysis of apoptosis inhibition

Cells were treated with 20 nmol/L FK866 alone or in combination with the pan-caspase inhibitor Z-VAD-FMK (20 μmol/L). Z-VAD-FMK was re-added every 24 hours in all experiments. At 72 hours, numbers of viable and dead cells were determined using the trypan blue exclusion method.

### Capillary-based immunoassays

Proteins were size-separated and immune-detected using an automated capillary–based immunoassay system (JESS Simple Western system from ProteinSimple), as described previously (21). Antibodies used were anti-NAMPT (Abcam, cat. #ab45890), anti-GAPDH (Cell Signaling Technology, cat. #2118, RRID: AB_561053), and anti-HSP60 (ProteinSimple, cat. #AF1800). Horseradish peroxidase (HRP)–conjugated anti–mouse Ig (ProteinSimple, cat. #042-205, RRID: AB_2860576) and anti–rabbit Ig-HRP (ProteinSimple, cat. #042-206, RRID: AB_2860577) were used as secondary antibodies for the JESS system.

### ^13^C-Glucose labeling experiments

EoL-1 cells were pretreated with or without 20 nmol/L FK866 in standard culture medium for 24 hours. The medium was then replaced with glucose-free RPMI-1640 medium (FUJIFILM Wako) supplemented with 10% dialyzed FCS and 2,000 mg/L [U-^13^C] glucose (Cambridge Isotope Laboratories). Cells were incubated in labeling medium for 0.5, 1, or 2 hours, with respective FK866 treatment conditions maintained throughout the labeling period. At indicated time points, cells were harvested and analyzed by ion chromatography–high-resolution mass spectrometry, as described previously (22). Metabolite levels were normalized to the protein content of each sample.

### Measurement of NAD using a colorimetric assay

Total NAD levels in cultured cells and tumor tissues were measured using an NAD^+^/NADH Quantification Kit (BioVision) according to the manufacturer’s instructions. Cell pellets and tumor tissues were collected, snap-frozen, and stored at −80°C until analysis. Samples were lysed, and protein concentrations were determined using a BCA Protein Assay Kit (Thermo Fisher Scientific). NAD levels were normalized to the protein content of each sample.

### Niacin rescue analysis

Cells were treated with 20 nmol/L FK866 alone or combined with 10 μmol/L NA, NAR, or NR. Viable cells were counted on day 4 using the trypan blue exclusion method and a hemocytometer. For NAD measurement, cells were collected at 24 hours and lysed. NAD levels were quantified using a NAD/NADH Assay Kit (DOJINDO Laboratories) according to the manufacturer’s instructions and normalized to total protein content.

### Metabolomic analysis

U937, MOLM-14, and EoL-1 cells were treated with 20 nmol/L FK866 or left untreated for 24 hours in triplicate. Cells were washed with ice-cold PBS, snap-frozen, and stored at −80°C until metabolite extraction. Global metabolomic profiling was performed by capillary electrophoresis–time-of-flight mass spectrometry (CE-TOFMS) as described previously (23, 24). Metabolites were identified and quantified based on migration time and accurate mass using standard CE-TOFMS workflows. Metabolite levels were normalized to total cellular protein content, as determined using a DC Protein Assay kit (Bio-Rad Laboratories, cat. #5000111).

Data analysis was performed as follows. First, metabolites that were not detected (ND) in >50% of samples (i.e., ≥10 of 18 samples across all conditions) were excluded from further analysis. For the remaining metabolites, ND values were imputed as one half of the minimum detected value across all samples. For each cell line, metabolite levels were averaged across biological triplicates. Differential analysis was performed across the three AML cell lines, with each treated as an independent sample. Energy charge was calculated using the formula: energy charge = [(XTP) + 0.5 × (XDP)]/[(XTP) + (XDP) + (XMP)], where XTP represents nucleoside triphosphates, XDP represents nucleoside diphosphates, XMP represents monophosphates, and X denotes either adenosine or guanosine.

### Mouse experiments

All animal experiments were performed with approval of the Miyagi Cancer Center Research Institute Animal Care and Use Committee. Mice were maintained in specific pathogen–free facilities with a 12-hour light–dark cycle, controlled temperature of ∼25°C, and controlled humidity of ∼50%. Unless stated otherwise, mice were fed a normal diet (MF; Oriental Yeast Co., Ltd.) *ad libitum*. A NA-free diet (NFD) was prepared by Oriental Yeast Co., Ltd. as described previously (16). NOD/Shi-scid, IL-2RγKO Jic mice (NOG; In-Vivo Science Inc., RRID: MGI:6197549) were used for xenograft experiments. Female C57BL/6NJcl mice (age: 8–10 weeks; CLEA Japan, Inc., RRID: IMSR_JCL:JCL:MIN-0004) were used for blood count analyses.

For EoL-1 and U937 AML models, NOG mice were conditioned with busulfan (1.33 mg/mL in 10% DMSO) administered as a single dose intraperitoneally at 10 mg/kg 24 hours before cell transplantation, as described previously (25). Cells (2 × 10^6^ and 5 × 10^6^ cells per mouse for U937-Luc and EoL-1-Luc cells, respectively) suspended in PBS were injected into NOG mice via the tail vein. Mice were randomly assigned to experimental groups, and treatment was initiated on day 5. GNE-617 [dissolved at 3 mg/mL in polyethylene glycol 400/H_2_O/EtOH (6:3:1) solution by sonication] was administered at 0.45 mg per mouse by oral gavage once a day for 4 consecutive days (at approximately 12:00 pm). The dose and treatment schedule were selected based on our previous studies (16, 18). Bioluminescence imaging of luciferase-expressing cells was performed under isoflurane anesthesia using an IVIS Lumina imaging system (PerkinElmer), as described previously (21). VivoGlo Luciferin (Promega) was administered intraperitoneally, and whole-body images were acquired 10 minutes after luciferin injection. Total photon flux (photons/second) was quantified using Living Image software (v4.5, PerkinElmer, RRID: SCR_014247). For survival analysis, an event was defined as death or euthanasia due to deterioration in the general condition, including marked body weight loss, reduced mobility, or inability to access food or water.

For MOLM-14 models, cells were suspended in a 1:1 mixture of Matrigel (Corning) and serum-free RPMI medium and injected into both flanks of NOG mice at 5 × 10^6^ cells per site subcutaneously. When tumors reached approximately 100 mm^3^, mice were assigned to treatment groups such that mean tumor volumes were comparable among groups at baseline. Tumor size was measured every 2 to 3 days using calipers, and tumor volume was calculated using the formula: (length × width^2^)/2.

For blood count tests, whole blood from female C57BL/6 mice (8–10 weeks) was collected into ethylenediaminetetraacetic acid (EDTA)-containing tubes and analyzed using an automated hematology analyzer (Sysmex). Following observation of treatment-induced leukopenia, blood counts were monitored longitudinally for up to 2 weeks after treatment to assess recovery.

### Flow cytometry analysis of splenic leukemic burden

Spleens were collected on day 15 after EoL-1-Luc cell transplantation and mechanically dissociated through a cell strainer to prepare single-cell suspensions. Following red blood cell (RBC) lysis, cells were stained with APC-conjugated anti-hCD45, FITC-conjugated anti-mCD45, and LIVE/DEAD Fixable Aqua Dead Cell Stain (Thermo Fisher Scientific). Data were acquired using an SA3800 flow cytometer (Sony) and analyzed using FlowJo software (Waters Biosciences; RRID: SCR_008520). After exclusion of debris, doublets, and dead cells, splenic leukemic burden was calculated as the percentage of hCD45^+^ cells among total CD45^+^ cells (hCD45^+^ plus mCD45^+^ cells).

### Histologic analysis of bone marrow and spleen

Femurs and spleens were collected at indicated time points and fixed overnight in 10% formalin prepared in PBS. Femurs were subsequently decalcified for 6 days using Decalcifying Solution B (EDTA formulation; FUJIFILM Wako). Tissues were then embedded in paraffin, sectioned, and stained with hematoxylin and eosin. Whole-slide images were acquired using a NanoZoomer digital slide scanner (Hamamatsu Photonics).

### Statistics and reproducibility

**Figure 1.**
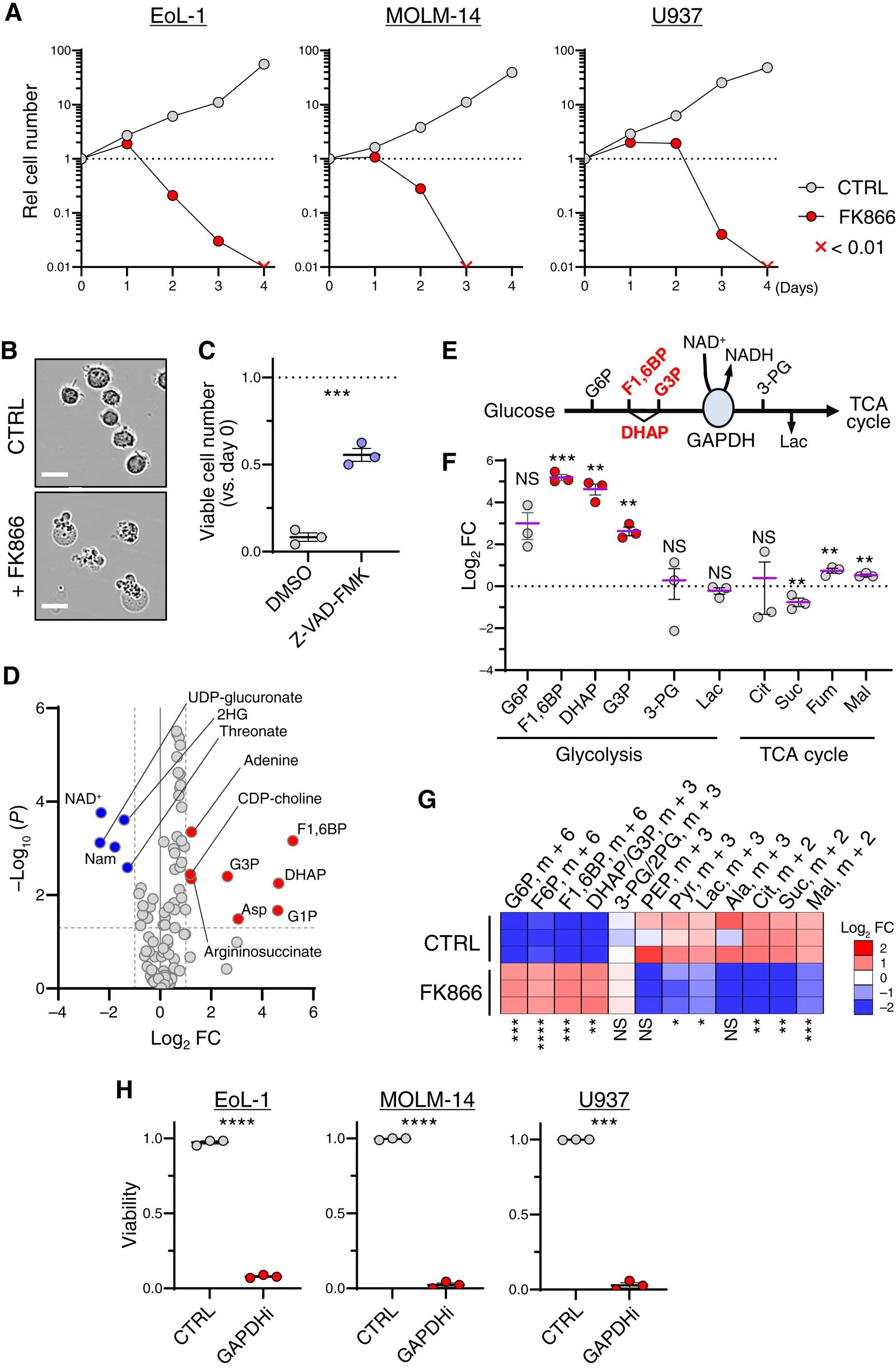
NAMPT inhibition perturbs glycolysis and induces apoptosis-like cell death in AML cells. **A,** Time course of FK866-induced cell death in the AML cell lines EoL-1, MOLM-14, and U937. Cells were cultured with or without 20 nmol/L FK866, and numbers of viable cells were counted over 4 days. Data are representative of two independent experiments and presented as the mean of four measurement replicates. Rel, relative. **B,** Apoptosis-like morphology of FK866-treated AML cells. Representative images of U937 cells at day 3 treated as in **A**. Scale bars, 20 μm. **C,** Effect of caspase inhibition on FK866-induced cell death. U937 cells were treated with FK866 alone or in combination with the pan-caspase inhibitor Z-VAD-FMK. Numbers of viable cells were assessed at day 3. *n* = 3 biological replicates. **D,** Volcano plot showing metabolomic changes following 24 hours of FK866 treatment of EoL-1, MOLM-14, and U937 cells. For each line, metabolite levels were averaged across biological triplicates. Differential analysis was performed across the three AML cell lines, each treated as an independent sample. Significantly increased and decreased metabolites are highlighted in red and blue, respectively. 2HG, 2-hydroxyglutarate; Asp, aspartate; G1P, glucose 1-phosphate; **E,** Schematic of glucose metabolism, highlighting the NAD^+^-dependent step catalyzed by GAPDH. The color code is the same as in **D**.** G6P, glucose 6-phosphate**; Lac, lactate. **F,** Relative changes in glycolytic and tricarboxylic acid (TCA) cycle metabolites following 24 hours of FK866 treatment of EoL-1, MOLM-14, and U937 cells. Metabolome analysis followed by differential analysis was performed as in **D**. Each symbol represents an individual cell line. FC, fold change. Cit, citrate; Fum, fumarate; Suc, succinate. **G,** Heatmap showing relative levels of ^13^C-isotopomers. EoL-1 cells were treated 24 hours with or without FK866 and then labeled with [^13^C-U] glucose for additional 2 hours. Each row represents an independent biological replicate; *n* = 3. 2PG, 2-phosphoglycerate; Ala, alanine; F6P, fructose 6-phosphate; Mal, malate; PEP, phosphoenolpyruvate; Pyr, pyruvate. **H,** Effects of GAPDH inhibition (GAPDHi) on AML cell survival. Cells were treated with 5 μmol/L KA, and cell viability was determined at 48 hours. Viability values were not normalized to untreated controls (CTRL). *n* = 3 biological replicates. In **C**, **F**, and **H**, data are presented as the mean of biological replicates + SEM. NS, not significant; *, *P* < 0.05; **, *P* < 0.01; ***, *P* < 0.001; ****, *P* < 0.0001, as determined by a two-tailed *t* test (**C**, **G**, and **H**) or one-sample *t* test (**F**).

No statistical methods were used to predetermine sample size. No data were excluded from analyses. Experiments were not randomized, and investigators were not blinded to allocation during experiments and outcome assessment. Two-tailed Student *t* tests and one-way ANOVA followed by the Tukey multiple comparisons test were used for comparisons between two groups and among multiple groups, respectively. A one-sample *t* test was used to determine whether normalized log_2_ fold changes differed significantly from zero in Fig. 1F. *P* < 0.05 was considered statistically significant. The numbers of biological replicates and independent experimental cohorts are specified in corresponding figure legends. Statistical analyses were performed using GraphPad Prism version 11.1.0 (GraphPad Software; RRID: SCR_002798).

## Results

### NAMPT inhibition induces GAPDH inactivation and apoptosis-like cell death in AML cells

To assess effects of NAMPT inhibition on AML cell proliferation and/or survival, we selected three cell lines EoL-1, MOLM-14, and U937 cells: MOLM-14 represents FAB-M5a AML with KMT2A::MLLT3 (formerly known as MLL-AF9), EoL-1 represents FIP1L1::PDGFRA-positive eosinophilic myeloid leukemia, and U937 is a histiocytic lymphoma–derived line with monocytic characteristics and a PICALM::MLLT10 fusion. We treated these lines with FK866, a small-molecule NAMPT inhibitor (NAMPTi), and counted viable cells up to day 4. FK866 induced robust cell death in all lines tested (Fig. 1A). FK866-treated cells exhibited morphologic features characteristic of apoptosis, including chromatin condensation and membrane blebbing (Fig. 1B; Supplementary Fig. S1A). Similar results were obtained in each line tested when we treated cells with a different NAMPTi, GNE-617 (Supplementary Fig. S1B). Both FK866 and GNE-617 inhibit NAMPT catalytic activity (7, 26), and FK866 treatment had minimal effects on NAMPT protein levels in EoL-1 and U937 cells (Supplementary Fig. S1C). FK866- or GNE-617–induced cell death in EoL-1 and U937 cells were almost completely blocked by the addition of exogenous NMN, an enzymatic product of NAMPT (Supplementary Fig. S1D–S1F). In U937 cells, pharmacologic inhibition of caspases with the pan-caspase inhibitor Z-VAD-FMK partially but significantly attenuated FK866-induced cell death (Fig. 1C). Collectively, these results indicate that NAMPT inhibition induces AML cell apoptosis.

Metabolome analysis of three AML lines (EoL-1, MOLM-14, and U937) demonstrated that NAD^+^ (the oxidized form of NAD) was the most significantly and markedly reduced metabolite following FK866 treatment (Fig. 1D), suggesting effective on-target inhibition of the NAD salvage pathway. Interestingly, levels of Nam, a salvage pathway substrate, were also significantly reduced in these FK866-treated AML cells (Fig. 1D). On the other hand, we observed robust accumulation of metabolites upstream of GAPDH in glycolysis, including fructose 1,6-bisphosphate (F1,6BP), dihydroxyacetone phosphate (DHAP), and G3P, in all AML lines after FK866 treatment, whereas levels of downstream GAPDH metabolites, such as 3-PG and lactate, and tricarboxylic acid cycle metabolites were largely unaffected (Fig. 1D–F). After FK866 treatment, all lines tested showed severely decreased NAD^+^ levels and marked accumulation of F1,6BP, DHAP, and G3P throughout the experimental time course (Supplementary Fig. S2A). Levels of other metabolites, such as 3-PG and citrate, varied depending on the time point and/or cell line. Tracer experiments using ^13^C-glucose demonstrated that FK866 treatment blocked glucose metabolism at or near the GAPDH-catalyzed step in EoL-1 cells (Fig. 1G; Supplementary Fig. S2B). Although reduced GAPDH activity could potentially alter cellular energy status, adenylate energy charge was not substantially decreased after 31 hours of FK866 treatment in EoL-1 and MOLM-14 cells or after 48 hours in U937 cells (Supplementary Fig. S2C). Given that GAPDH is NAD^+^ dependent (Fig. 1E; refs. 27–29), these results strongly suggest that decreased NAD levels due to NAMPT inhibition results in GAPDH inactivation in AML cells.

We then hypothesized that downregulation of GAPDH activity would induce cell death. To test this possibility, we performed pharmacologic experiments using a GAPDH inhibitor, koningic acid (KA). KA, which inhibits GAPDH catalytic activity (30), had minimal effects on GAPDH protein levels in EoL-1 and U937 cells (Supplementary Fig. S3A). Metabolome analyses revealed that KA treatment led to robust accumulation of glycolytic intermediates upstream of GAPDH in EoL-1 cells (Supplementary Fig. S3B). Levels of some downstream metabolites in these cells, including 3-PG, also increased at 20 hours, suggesting that KA-mediated metabolic changes are complex particularly at later time points (Supplementary Fig. S3B). Importantly, KA treatment induced robust cell death in all three lines tested (Fig. 1H), as well as morphologic features of apoptosis (data not shown). Moreover, the addition of 3-PG, a metabolite downstream of GAPDH, rescued EoL-1 and U937 cells from cell death induced by FK866, whereas G3P, a GAPDH substrate, did not (Supplementary Fig. S3C and S3D). These results overall suggest that NAMPT inhibition induces AML cell apoptosis in part through GAPDH inactivation.

### Exogenous NA and NAR bypass NAMPT inhibition–induced AML cell death

**Figure 2.**
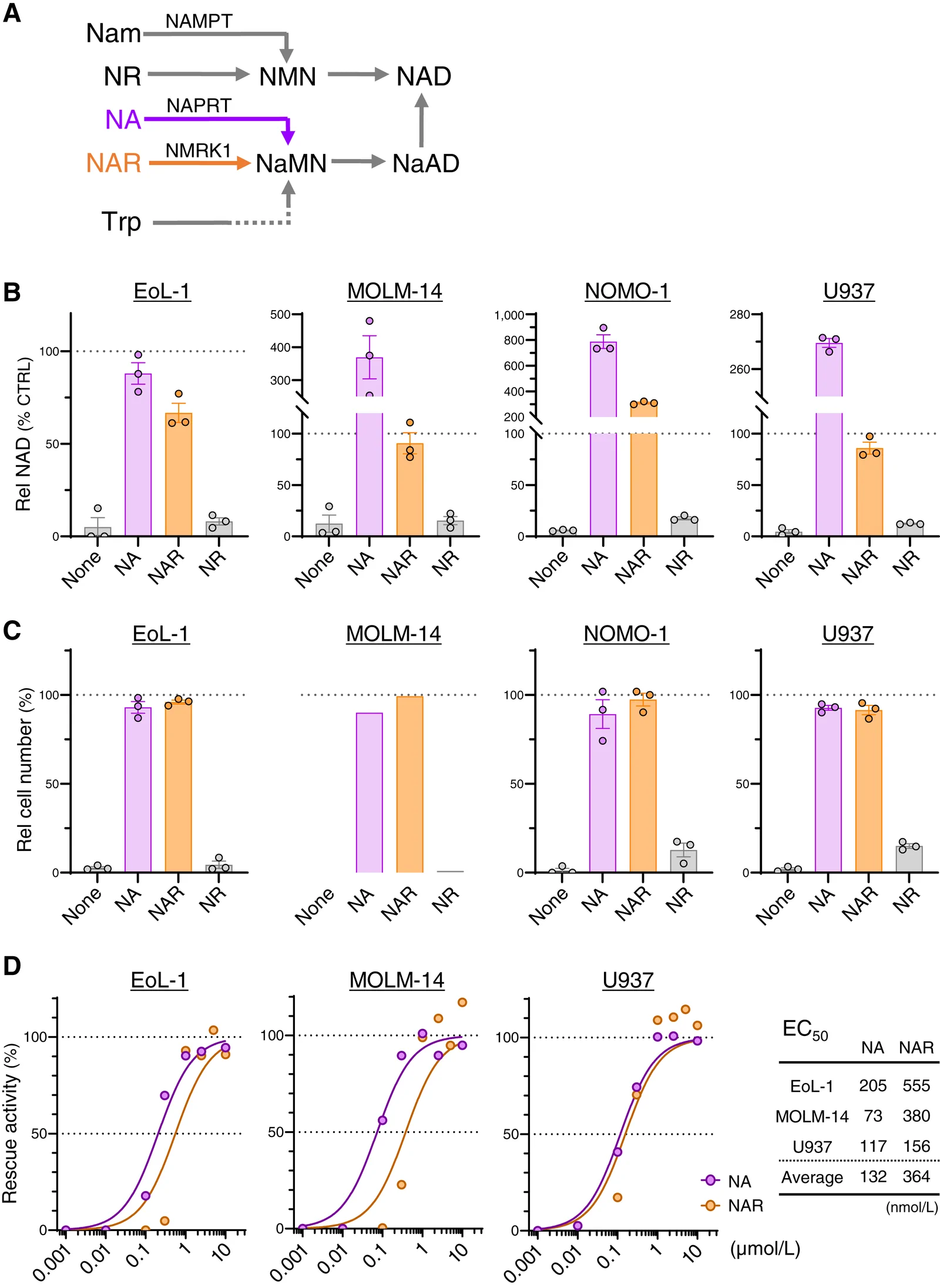
Exogenous NA and NAR rescue AML cells from NAMPT inhibition. **A,** Schematic showing NAD biosynthesis pathways in vertebrates. NaAD, NA adenine dinucleotide; NaMN, NA mononucleotide. NAMPT, nicotinic acid phosphoribosyltransferase (NAPRT), and NMRK1 are key enzymes in the corresponding pathway. **B,** NAD levels in AML cells treated 24 hours with FK866 in the presence or absence of 10 μmol/L NA, NAR, or NR. Results are shown relative to untreated controls (CTRL), which were defined as 100%. *n* = 3 biological replicates. Rel, relative. **C,** Viable numbers of AML cells treated as in **B** for 4 days. Results are shown relative to untreated controls, which were defined as 100%. *n* = 3 biological replicates. **D,** Rescue experiments using various concentrations of NR, NAR, or NA in cells treated 4 days with 20 nmol/L FK866. The number of viable cells was determined, and rescue was quantified using the formula: rescue activity = [(number of cells treated with FK866 plus niacin) − (number of cells treated with FK866 alone)]/[(number of untreated cells) − (number of cells treated with FK866 alone)]. Representative data from two independent experiments. Data are presented as the mean of biological replicates + SEM (**B** and **C**).

As shown above (see Fig. 1A), NAMPT inhibition induced robust cell death in AML cells under standard culture conditions. Relevant to factors required for NAD biosynthesis (summarized in Fig. 2A), standard culture media contain abundant Nam and Trp but lack other NAD precursors, such as NA, NAR, or NR, which are present in physiologic environments, and many of the latter are known to attenuate effects of NAMPT inhibition *in vivo* (16, 31). Thus, we asked whether AML cells can synthesize NAD using alternative precursors after NAMPT inhibition. To do so, we first treated four AML lines, including an additional line (NOMO-1, established from FAB-M5a AML), with FK866 in the presence of exogenous NA, NAR, or NR at supraphysiologic concentrations (10 μmol/L) and then measured cellular NAD levels. NA or NAR supplementation potently restored NAD levels following FK866 treatment in all AML cell lines tested (Fig. 2B). In MOLM-14, NOMO-1, and U937 lines, exogenous NA or NAR increased NAD abundance to levels markedly exceeding basal levels (Fig. 2B). In contrast, exogenous 10 μmol/L NR did not increase NAD levels in any FK866-treated AML cells tested (Fig. 2B). Consistently, supplementation with NA or NAR, but not NR, largely decreased cell death induced by FK866 (Fig. 2C). These results demonstrate that in the AML cell lines tested, NA or NAR can bypass NAMPTi-induced cell death when supplied at sufficient concentrations.

We previously reported that blood concentrations of NA, NAR, and NR in mice are approximately 10, 600, and 500 nmol/L, respectively (16). Given that NA and NAR can rescue AML cells from FK866 toxicity, we performed dose–response experiments with NA and NAR to determine concentrations required for rescue. EC_50_ values (defined as concentrations of exogenous niacin required for 50% rescue from FK866 toxicity) were ∼132 nmol/L for NA and ∼364 nmol/L for NAR (Fig. 2D). Overall, these results indicate that NAR can bypass FK866 toxicity at near-physiologic concentrations, whereas NA-mediated rescue requires supraphysiologic concentrations (∼13-fold above physiologic levels).

### Restriction of dietary NA boosts NAMPTi efficacy in AML xenograft models

**Figure 3.**
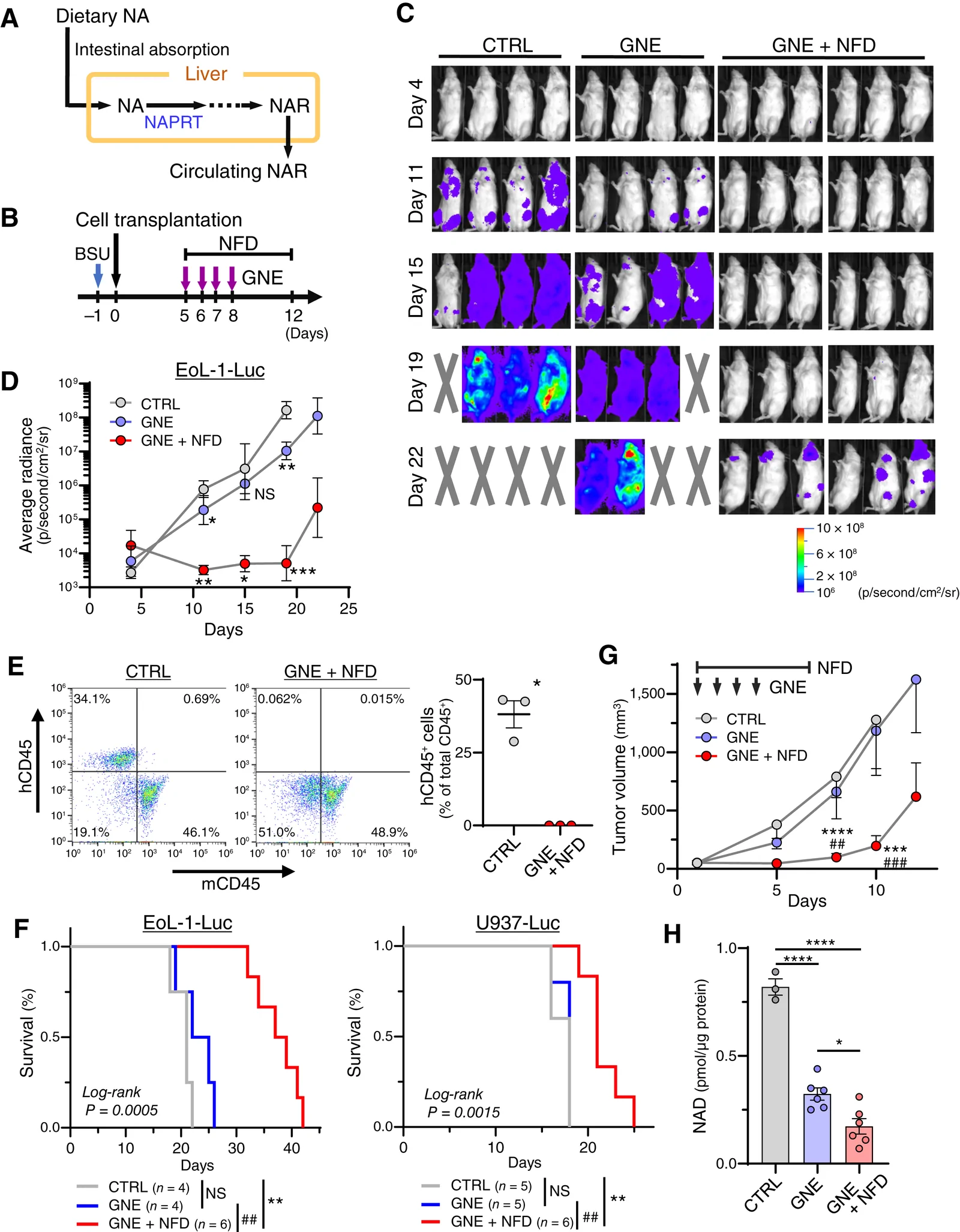
Restriction of dietary NA boosts NAMPTi efficacy in AML xenograft models. **A,** Schematic showing conversion of dietary NA into circulating NAR via host metabolism. **B,** Experimental design of intravenous transplantation models. Mice fed a normal diet were conditioned with busulfan (BSU) on day −1 and inoculated with luciferase-labeled AML cells on day 0. On day 5, GNE-617 (GNE) treatment was initiated in all mice, and half of the mice remained on a normal diet. The other half (GNE + NFD) were switched to an NFD. Control (CTRL) mice were not treated with GNE and remained on a normal diet for the entire experiment. **C,** Bioluminescence imaging of EoL-1-Luc–transplanted mice on indicated days. As in **B**, CTRL mice were fed a normal diet and were vehicle-treated, GNE mice were fed a normal diet and treated with GNE, and GNE + NFD mice were treated with GNE and fed an NFD. **D,** Quantification of leukemic burden based on bioluminescent imaging in groups described in **C** over time (days 4–22). **E,** Splenic leukemic burden in EoL-1-Luc xenograft mice on day 15. Spleens were collected from CTRL and GNE + NFD-treated mice on day 15. Representative flow cytometry plots (left) and quantification of hCD45^+^ leukemia cells among total CD45^+^ splenocytes (right). Each symbol represents an individual mouse. *n* = 3 mice per group. **F,** Survival of mice transplanted intravenously with EoL-1-Luc or U937-Luc cells and treated using the same regimen described in **C**. **G,** Tumor volumes of MOLM-14 subcutaneous xenografts. Mice were inoculated subcutaneously with MOLM-14 cells on day 0 and treated as in **C**. *n* = 6 tumors in each group. **H,** NAD levels in subcutaneous EoL-1-Luc tumors from mice treated with GNE alone or in combination with NFD. Mice received a single dose of GNE at 0 hour and were maintained on a normal diet or an NFD until tumor collection at 27 hours. CTRL mice received vehicle and the normal diet. Each symbol represents one tumor. *n* = 3 (CTRL) or 6 (GNE and GNE + NFD) tumors. Data are presented as mean + SEM (**D**, **E**, **G**, and **H**). NS, not significant; *, *P* < 0.05; **, *P* < 0.01; ***, *P* < 0.001; ****, *P* < 0.0001 vs. CTRL, and ^##^, *P* < 0.01; ^###^, *P* < 0.001 vs. GNE, determined by one-way ANOVA with a *post hoc* test (**D**, **G**, and **H**), a two-tailed unpaired *t* test (**E**), or a log-rank test (**F**).

We next asked whether modulating circulating NAR levels would alter NAMPTi efficacy in AML xenograft models. Circulating NAR is synthesized nicotinic acid phosphoribosyltransferase dependently from dietary NA (Fig. 3A; refs. 16, 17); thus, NA restriction reduces circulating NAR levels. We previously showed that eliminating dietary NA markedly reduced circulating NAR levels in mice, with little effect on NA levels (16). Thus, we asked whether comparable dietary intervention would enhance NAMPT inhibition in AML xenograft models. To do so, we inoculated NOG mice intravenously with luciferase-labeled EoL-1 cells and then 5 days later divided mice into three groups: one treated with the NAMPTi GNE-617 and fed a normal diet; another comparably treated with GNE-617 but fed an NFD (Fig. 3B); and a control group fed a normal diet and treated with vehicle only. The GNE-617 dose and treatment schedule were selected based on our previous studies (16, 18). Compared with both the control and GNE-617/normal diet groups, GNE-617–treated mice fed the NFD (GNE-617 + NFD) showed significantly suppressed tumor burden (Fig. 3C and D). At day 15, bone marrow (BM) in control mice was extensively infiltrated by blasts, with marked loss of normal hematopoietic elements (Supplementary Fig. S4A). In contrast, normal marrow morphology was largely preserved after combined GNE-617–NFD treatment (Supplementary Fig. S4A). At the same time point, hCD45^+^ leukemia cells were abundant in spleens of control mice but nearly absent after combined treatment (Fig. 3E). For survival analyses, an event was defined as death or euthanasia due to deterioration in the general condition. GNE-617 + NFD treatment significantly prolonged overall survival (Fig. 3F), whereas effects of NFD alone on the tumor burden and mouse survival were minimal (Supplementary Fig. S4B and S4C). Similar results were seen in the U937 leukemic model (Fig. 3F; Supplementary Fig. S4D–S4G) and in a subcutaneous MOLM-14 xenograft model (Fig. 3G). Treatment of mice with GNE-617 significantly decreased NAD levels in tumor cells, an activity further enhanced in combination with NFD in a subcutaneous EoL-1 xenograft model (Fig. 3H). Taken together, these results demonstrate that restriction of dietary NA significantly enhances NAMPTi therapeutic efficacy against AML in mice.

### Tolerability and hematologic effects of NAMPTi combined with NA restriction

**Figure 4.**
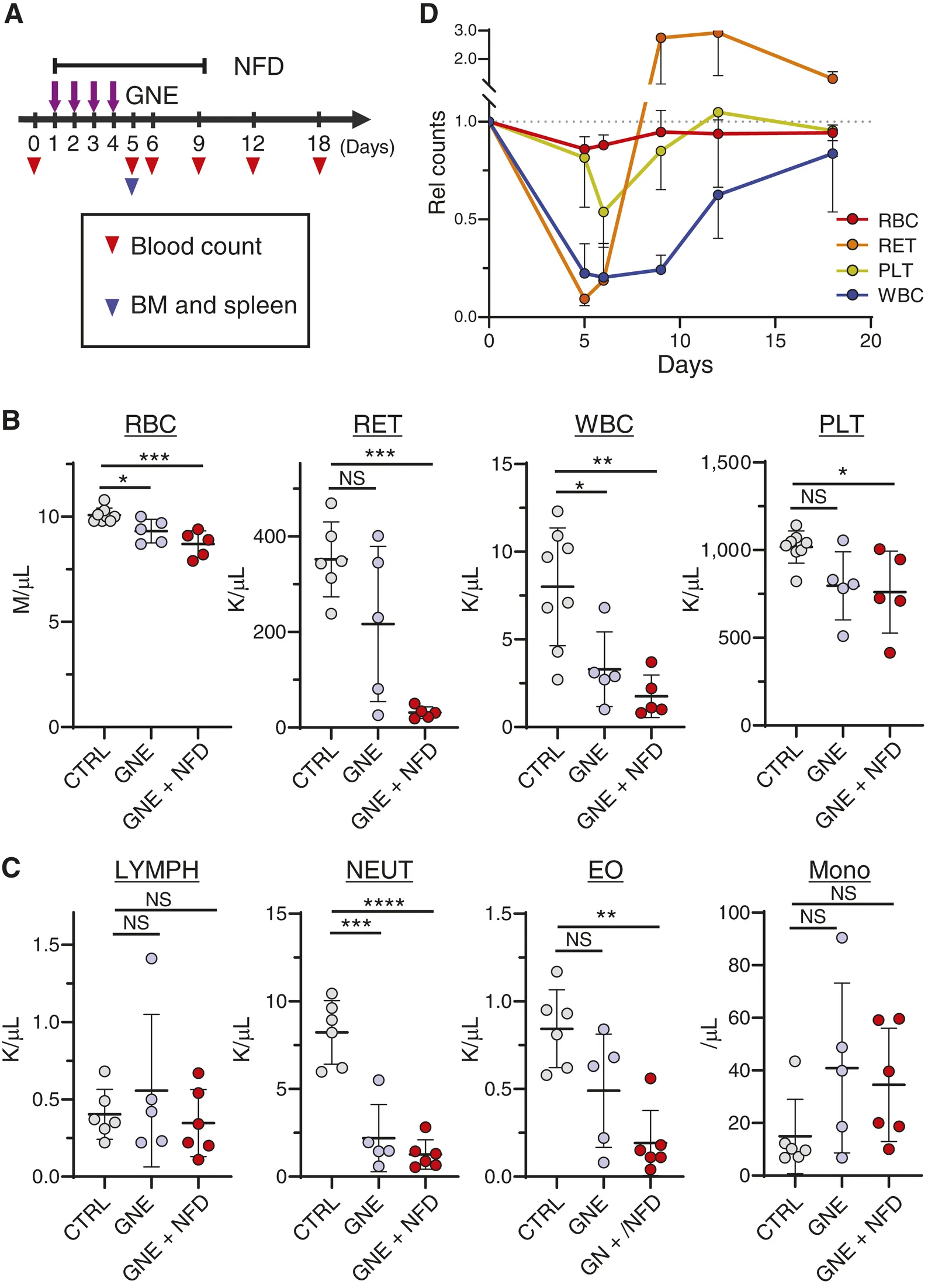
Tolerability and hematologic effects of NAMPTi combined with NA restriction. **A,** Experimental design for the toxicity assessment. C57BL/6 mice were assigned to three groups: control mice treated with vehicle and maintained on a normal diet (CTRL), GNE-617–treated mice maintained on a normal diet (GNE), and GNE-617–treated mice maintained on NFD (GNE + NFD). Mice in the GNE + NFD group were switched to NFD 1 day prior to the first GNE-617 administration, maintained on an NFD through day 9, and then returned to the normal diet. GNE-617 was administered once daily on days 1 to 4. Time points for peripheral blood counts and collection of BM and spleen are also indicated. **B,** Peripheral blood counts of RBCs, RET, WBCs, and PLT in mice treated with vehicle (CTRL) or GNE-617 and fed either a normal diet (GNE) or an NFD (GNE + NFD). Data for day 5. *n* = 5–8 mice. **C,** Peripheral blood counts of lymphocytes (LYMPH), NEUTs, EOs, and monocytes (Mono) in the same mice as in **B**. **D,** Time course of blood counts following treatment with GNE-617 + NFD. Data were normalized to day 0. *n* = 5–8 mice. Data are presented as the mean of biological replicates ± SD. NS, not significant; *, *P* < 0.05; **, *P* < 0.01; ***, *P* < 0.001; ****, *P* < 0.0001 as determined by one-way ANOVA with a *post hoc* test (**B** and **C**). Rel, relative.

We recently reported that mice treated with GNE-617 and fed an NFD showed no overt toxicity; they did not exhibit abnormalities in liver and kidney function, muscle integrity, or blood glucose levels (18). However, several NAMPTis, including FK866, GNE-617, and GMX-1778, reportedly cause thrombocytopenia and myelosuppression (32–35), prompting us to evaluate the hematologic toxicity of GNE-617 alone and in combination with NFD in C57BL/6 mice (Fig. 4A). GNE-617 treatment alone reduced or tended to reduce counts of immature and mature RBCs [reticulocytes (RET) and RBC], white blood cells (WBC), and platelets (PLT); these effects were enhanced by NA restriction (Fig. 4B), with RET and WBC showing the most pronounced reductions. Among WBCs, GNE-617 + NFD reduced the number of neutrophils (NEUT) and eosinophils (EO) but had no significant effects on lymphocyte and monocyte counts (Fig. 4C). For histologic analyses, we collected femoral BM and spleen at day 5, when reductions in peripheral WBC and RET counts were pronounced. Those analyses revealed that combined GNE-617 and NFD treatment decreases the number of nonnucleated erythroid cells in BM, although its effects on myeloid cells were minimal (Supplementary Fig. S5A). No overt histopathologic damage was seen in the spleen of GNE-617/NFD-treated mice (Supplementary Fig. S5B); both the splenic architecture and white pulp were well preserved. By day 18, most cell counts had largely returned to baseline levels (Fig. 4D; Supplementary Fig. S5C). Notably, we observed a transient rebound in RET counts during the recovery phase, indicating robust hematopoietic recovery, whereas NEUT and EO recovery remained partial (Fig. 4D; Supplementary Fig. S5C). Overall, the transience and reversibility of these hematologic effects support the tolerability of this treatment, which results in a survival benefit in multiple AML mouse models.

## Discussion

In this study, we demonstrate that AML cell lines can utilize NAR to maintain NAD levels and survive under NAMPT inhibition *in vitro*. Furthermore, we report that NA restriction, which lowers circulating NAR levels, enhances antitumor efficacy of NAMPT inhibition in AML xenograft models. Previous analyses of *ex vivo* cultured primary AML cells show that the LSC fraction exhibits significantly higher sensitivity to NAMPT inhibition than the non-LSC fraction, although underlying mechanisms remain controversial (19, 20). Here, we show that AML cell lines also display significant vulnerability to NAMPT inhibition. Notably, enhanced NAD biosynthesis through the salvage pathway in the AML LSC fraction has been linked to venetoclax resistance (20). Although it is not yet clear whether the LSC population can metabolize NAR, targeting NAR-dependent NAD biosynthesis may warrant further investigation as a strategy to overcome venetoclax resistance, a clinically relevant challenge in AML treatment (36–40). Our findings suggest that dietary intervention may represent one such approach to achieve this (41); we observed that combining an NAMPTi with NA restriction provided therapeutic benefits and was tolerated in mice.

Specifically, NAMPT inhibition induced apoptosis-like cell death in AML cells. Our metabolic studies strongly suggest that NAMPT inhibition reduces GAPDH activity in AML cells and that reduced GAPDH activity, in turn, promotes AML cell death. Lowered GAPDH activity would be expected to compromise cellular energy status (42, 43). However, we observed no substantial decrease in energy charge at the latest time points examined (31 hours in EoL-1 and MOLM-14 cells and 48 hours in U937 cells). Nevertheless, deterioration of cellular energy status at later time points cannot be excluded. Furthermore, because numerous NAD-dependent enzymes, including metabolic enzymes, PARPs, and sirtuins, require NAD for activity (1–3), reduced activity of these factors may also contribute to apoptosis induction following NAMPT inhibition.

We observed that NAD levels were severely reduced in AML cells following NAMPT inhibition, indicating that NAD biosynthesis via the *de novo* pathway, which utilizes Trp as a substrate, is insufficient to compensate for NAMPT inhibition. These findings are consistent with previous reports by Jones and colleagues (20). However, rescue experiments indicate that NAD biosynthesis from NA and NAR, which converges at the level of NA mononucleotide downstream of the *de novo* pathway, is sufficient to allow AML cell survival. In contrast to NA and NAR, NR showed minimal ability to rescue FK866-induced cytotoxicity at concentrations tested. In our previous study, NR rescued cells from the FK866 toxicity at submillimolar concentrations, albeit in a different cell type (16). Thus, NR may have substantially lower rescue potency than NA or NAR, possibly due to limited cellular uptake and/or nicotinamide riboside kinase (NMRK)-dependent phosphorylation. Whether NR utilization is particularly limited in AML cells requires further investigation. We observed similar rescue activities of NA and NAR against NAMPT inhibition *in vitro*, with comparable EC_50_ values. Thus, NA could, in principle, contribute to NAD maintenance under NAMPT inhibition *in vivo*. However, circulating NA levels are substantially lower than those of NAR and are not altered by dietary restriction (16), arguing against a major contribution of NA in this context. Notably, AML LSCs reportedly have limited capacity to utilize NA for NAD biosynthesis, whereas normal HSCs retain this capacity (19). In contrast, dietary NA restriction markedly reduces circulating NAR levels (16). Together with our *in vitro* findings that NAR effectively supports NAD maintenance under NAMPT inhibition, these observations suggest a model in which circulating NAR contributes to NAD maintenance *in vivo*. Determining the extent to which NAR contributes to NAD maintenance and therapeutic response *in vivo* will require genetic disruption of NAR metabolism, for example, by targeting NMRK1/2 (44–46).

In the present study, GNE-617 monotherapy reduced tumor NAD levels but had little effect on tumor growth and mouse survival. We selected the dose and treatment schedule based on previous studies in which comparable regimens showed substantial antitumor effects (16, 18), but we did not determine the maximum tolerated dose or systematically optimize the dose, schedule, or route of administration in mice. Because NAMPTi efficacy reportedly depends on dosing schedule and frequency (11, 31), further optimization might enhance antitumor efficacy of GNE-617 monotherapy. It is also possible that other NAMPTis may have greater monotherapeutic efficacy in these models. Although direct comparisons are difficult because of different models and the specific NAMPTi used, previous studies report substantial antitumor effects of NAMPTi monotherapy (11, 47). Whether dietary NA restriction provides an additional therapeutic benefit under such conditions remains to be determined.

Finally, we assessed potential toxicity of NAMPTi, with or without NA restriction, on normal hematopoiesis in mice and observed a transient decrease in blood counts of RBCs, WBCs, and PLT. However, all blood cell counts recovered to baseline levels within 10 days after the final dose. Nahimana and colleagues (48) have reported that hematologic toxicity, particularly leukopenia, represents a major adverse effect of NAMPT inhibition. Although previous studies have shown that NA supplementation can mitigate the hematologic toxicity of NAMPTis (32, 49), NA supplementation also reportedly attenuates NAMPTi antitumor efficacy in mice (11, 31). Our finding that NA restriction enhanced both toxicity and antitumor efficacy of NAMPTis is consistent with these previous studies. Subedi and colleagues (19) reported that AML LSCs are vulnerable to NAMPT inhibition, whereas normal HSCs do not exhibit comparable sensitivity, an observation consistent with the transient nature of leukopenia reported here. Although careful monitoring and management of leukopenia may be required, these findings collectively indicate that the treatment is generally well tolerated in mice.

In summary, our study shows that AML cells utilize NAR to sustain NAD under NAMPT inhibition and that dietary NA restriction, which selectively lowers circulating NAR levels, enhances NAMPTi efficacy in mice. These findings highlight the importance of systemic NAD precursor metabolism, including noncanonical niacin species such as NAR, in shaping responses to NAMPT-targeted therapies currently being evaluated in clinical trials (50, 51).

## Supplementary Material

Supplementary Figure S1 Figure S1. Effects of NAMPTi on AML cell proliferation, related to Fig. 1.

Supplementary Figure S2 Figure S2. Effects of FK866 on AML cell metabolism, related to Fig. 1.

Supplementary Figure S3 Figure S3. Effects of KA and exogenous 3-PG, related to Fig. 1.

Supplementary Figure S4 Figure S4. Effects of NAMPTi and NFD on leukemic burden, related to Fig. 3.

Supplementary Figure S5 Figure S5. Effects of NAMPTi and NFD on hematopoiesis, related to Fig. 4.

Supplementary Table 1 Table S1. Metabolomics data from AML cell lines treated with FK866

Supplementary Data 1 Supplementary Data 1

## Data Availability

The metabolomic data generated in this study are provided in Supplementary Table S1. Source data are provided with this article as a Source Data file. All other data are available upon request to the corresponding author.

## References

[1] Katsyuba E , Romani M, Hofer D, Auwerx J. NAD+ homeostasis in health and disease. Nat Metab 2020;2:9–31. 10.1038/s42255-019-0161-5. 32694684

[2] Chiarugi A , Dölle C, Felici R, Ziegler M. The NAD metabolome—a key determinant of cancer cell biology. Nat Rev Cancer 2012;12:741–52. 10.1038/nrc3340. 23018234

[3] Migaud ME , Ziegler M, Baur JA. Regulation of and challenges in targeting NAD+ metabolism. Nat Rev Mol Cell Biol 2024;25:822–40. 10.1038/s41580-024-00752-w. PMC12456757 39026037

[4] Liu L , Su X, Quinn WJ 3rd, Hui S, Krukenberg K, Frederick DW, . Quantitative analysis of NAD synthesis–breakdown fluxes. Cell Metab 2018;27:1067–80.e5. 10.1016/j.cmet.2018.03.018. PMC5932087 29685734

[5] Heske CM . Beyond energy metabolism: exploiting the additional roles of NAMPT for cancer therapy. Front Oncol 2020;9:1514. 10.3389/fonc.2019.01514. PMC6978772 32010616

[6] Galli U , Colombo G, Travelli C, Tron GC, Genazzani AA, Grolla AA. Recent advances in NAMPT inhibitors: a novel immunotherapic strategy. Front Pharmacol 2020;11:656. 10.3389/fphar.2020.00656. PMC7235340 32477131

[7] Hasmann M , Schemainda I. FK866, a highly specific noncompetitive inhibitor of nicotinamide phosphoribosyltransferase, represents a novel mechanism for induction of tumor cell apoptosis. Cancer Res 2003;63:7436–42. 14612543

[8] Liederer BM , Cheong J, Chou KJ, Dragovich PS, Le H, Liang X, . Preclinical assessment of the ADME, efficacy and drug–drug interaction potential of a novel NAMPT inhibitor. Xenobiotica 2019;49:1063–77. 10.1080/00498254.2018.1528407. 30257601

[9] Cea M , Cagnetta A, Fulciniti M, Tai YT, Hideshima T, Chauhan D, . Targeting NAD+ salvage pathway induces autophagy in multiple myeloma cells via mTORC1 and extracellular signal-regulated kinase (ERK1/2) inhibition. Blood 2012;120:3519–29. 10.1182/blood-2012-03-416776. PMC3482862 22955917

[10] Audrito V , Managò A, La Vecchia S, Zamporlini F, Vitale N, Baroni G, . Nicotinamide phosphoribosyltransferase (NAMPT) as a therapeutic target in BRAF-mutated metastatic melanoma. J Natl Cancer Inst 2018;110:djx198. 10.1093/jnci/djx198. 29309612

[11] Korotchkina L , Kazyulkin D, Komarov PG, Polinsky A, Andrianova EL, Joshi S, . OT-82, a novel anticancer drug candidate that targets the strong dependence of hematological malignancies on NAD biosynthesis. Leukemia 2020;34:1828–39. 10.1038/s41375-019-0692-5. PMC7326709 31896781

[12] Abu Aboud O , Chen C-H, Senapedis W, Baloglu E, Argueta C, Weiss RH. Dual and specific inhibition of NAMPT and PAK4 by KPT-9274 decreases kidney cancer growth. Mol Cancer Ther 2016;15:2119–29. 10.1158/1535-7163.MCT-16-0197. PMC5010932 27390344

[13] Piacente F , Caffa I, Ravera S, Sociali G, Passalacqua M, Vellone VG, . Nicotinic acid phosphoribosyltransferase regulates cancer cell metabolism, susceptibility to NAMPT inhibitors, and DNA repair. Cancer Res 2017;77:3857–69. 10.1158/0008-5472.CAN-16-3079. 28507103

[14] Kudo K , Nomura M, Sakamoto Y, Ito S, Morita M, Kawai M, . Divergent metabolic responses dictate vulnerability to NAMPT inhibition in ovarian cancer. FEBS Lett 2020;594:1379–88. 10.1002/1873-3468.13736. 31950503

[15] Chowdhry S , Zanca C, Rajkumar U, Koga T, Diao Y, Raviram R, . NAD metabolic dependency in cancer is shaped by gene amplification and enhancer remodelling. Nature 2019;569:570–5. 10.1038/s41586-019-1150-2. PMC7138021 31019297

[16] Nomura M , Ohuchi M, Sakamoto Y, Kudo K, Yaku K, Soga T, . Niacin restriction with NAMPT inhibition is synthetic lethal to neuroendocrine carcinoma. Nat Commun 2023;14:8095. 10.1038/s41467-023-43630-3. PMC10719245 38092728

[17] Song WS , Shen X, Du K, Ramirez CB, Park SH, Cao Y, . Nicotinic acid riboside maintains NAD^+^ homeostasis and ameliorates aging-associated NAD^+^ decline. Cell Metab 2025;37:1499–514.e4. 10.1016/j.cmet.2025.05.004. PMC12328062 40315855

[18] Tsurumi K , Nomura M, Ouchi M, Yagishita S, Kikuchi N, Sato T, . Nicotinic acid restriction enhances the therapeutic benefit of NAMPT inhibition in small-cell lung cancer models. Cancer Sci 2026 Jul 28 [Epub ahead of print]. 10.1111/cas.70486. PMC13413092 42521252

[19] Subedi A , Liu Q, Ayyathan DM, Sharon D, Cathelin S, Hosseini M, . Nicotinamide phosphoribosyltransferase inhibitors selectively induce apoptosis of AML stem cells by disrupting lipid homeostasis. Cell Stem Cell 2021;28:1851–67.e8. 10.1016/j.stem.2021.06.004. 34293334

[20] Jones CL , Stevens BM, Pollyea DA, Culp-Hill R, Reisz JA, Nemkov T, . Nicotinamide metabolism mediates resistance to venetoclax in relapsed acute myeloid leukemia stem cells. Cell Stem Cell 2020;27:748–64.e4. 10.1016/j.stem.2020.07.021. PMC7655603 32822582

[21] Hashimoto E , Ohuchi M, Nomura M, Miyahara S, Hayashi K, Saito M, . The iron–sulfur cluster assembly factor FDX2 is required for tumor initiation but not for growth of established tumors in transplantation models. J Biol Chem 2026;302:113200. 10.1016/j.jbc.2026.113200. PMC13284478 42208897

[22] Maeda R , Seki N, Uwamino Y, Wakui M, Nakagama Y, Kido Y, . Amino acid catabolite markers for early prognostication of pneumonia in patients with COVID-19. Nat Commun 2023;14:8469. 10.1038/s41467-023-44266-z. PMC10733290 38123556

[23] Morita M , Sato T, Nomura M, Sakamoto Y, Inoue Y, Tanaka R, . PKM1 confers metabolic advantages and promotes cell-autonomous tumor cell growth. Cancer Cell 2018;33:355–67.e7. 10.1016/j.ccell.2018.02.004. 29533781

[24] Miyahara S , Ohuchi M, Nomura M, Hashimoto E, Soga T, Saito R, . FDX2, an iron–sulfur cluster assembly factor, is essential to prevent cellular senescence, apoptosis or ferroptosis of ovarian cancer cells. J Biol Chem 2024;300:107678. 10.1016/j.jbc.2024.107678. PMC11414659 39151727

[25] Saland E , Boutzen H, Castellano R, Pouyet L, Griessinger E, Larrue C, . A robust and rapid xenograft model to assess efficacy of chemotherapeutic agents for human acute myeloid leukemia. Blood Cancer J 2015;5:e297. 10.1038/bcj.2015.19. PMC4382660 25794133

[26] Zheng X , Bair KW, Bauer P, Baumeister T, Bowman KK, Buckmelter AJ, . Identification of amides derived from 1H-pyrazolo[3,4-b]pyridine-5-carboxylic acid as potent inhibitors of human nicotinamide phosphoribosyltransferase (NAMPT). Bioorg Med Chem Lett 2013;23:5488–97. 10.1016/j.bmcl.2013.08.074. 24021463

[27] Velick SF . Coenzyme binding and the thiol groups of glyceraldehyde-3-phosphate dehydrogenase. J Biol Chem 1953;203:563–73. 10.1016/S0021-9258(19)52326-2. 13084626

[28] Kumagai S , Narasaki R, Hasumi K. Glucose-dependent active ATP depletion by koningic acid kills high-glycolytic cells. Biochem Biophys Res Commun 2008;365:362–8. 10.1016/j.bbrc.2007.10.199. 17997978

[29] Tan B , Dong S, Shepard RL, Kays L, Roth KD, Geeganage S, . Inhibition of nicotinamide phosphoribosyltransferase (NAMPT), an enzyme essential for NAD+ biosynthesis, leads to altered carbohydrate metabolism in cancer cells. J Biol Chem 2015;290:15812–24. 10.1074/jbc.M114.632141. PMC4505489 25944913

[30] Kato M , Sakai K, Endo A. Koningic acid (heptelidic acid) inhibition of glyceraldehyde-3-phosphate dehydrogenases from various sources. Biochim Biophys Acta 1992;1120:113–6. 10.1016/0167-4838(92)90431-c. 1554737

[31] O’Brien T , Oeh J, Xiao Y, Liang X, Vanderbilt A, Qin A, . Supplementation of nicotinic acid with NAMPT inhibitors results in loss of in vivo efficacy in NAPRT1-deficient tumor models. Neoplasia 2013;15:1314–29. 10.1593/neo.131718. PMC3884523 24403854

[32] Tarrant JM , Dhawan P, Singh J, Zabka TS, Clarke E, DosSantos G, . Preclinical models of nicotinamide phosphoribosyltransferase inhibitor-mediated hematotoxicity and mitigation by co-treatment with nicotinic acid. Toxicol Mech Methods 2015;25:201–11. 10.3109/15376516.2015.1014080. 25894564

[33] Holen K , Saltz LB, Hollywood E, Burk K, Hanauske AR. The pharmacokinetics, toxicities, and biologic effects of FK866, a nicotinamide adenine dinucleotide biosynthesis inhibitor. Invest New Drugs 2008;26:45–51. 10.1007/s10637-007-9083-2. 17924057

[34] Hovstadius P , Larsson R, Jonsson E, Skov T, Kissmeyer AM, Krasilnikoff K, . A phase I study of CHS 828 in patients with solid tumor malignancy. Clin Cancer Res 2002;8:2843–50. 12231525

[35] von Heideman A , Berglund A, Larsson R, Nygren P. Safety and efficacy of NAD depleting cancer drugs: results of a phase I clinical trial of CHS 828 and overview of published data. Cancer Chemother Pharmacol 2010;65:1165–72. 10.1007/s00280-009-1125-3. 19789873

[36] Maiti A , Rausch CR, Cortes JE, Pemmaraju N, Daver NG, Ravandi F, . Outcomes of relapsed or refractory acute myeloid leukemia after frontline hypomethylating agent and venetoclax regimens. Haematologica 2021;106:894–8. 10.3324/haematol.2020.252569. PMC7927994 32499238

[37] DiNardo CD , Pratz K, Pullarkat V, Jonas BA, Arellano M, Becker PS, . Venetoclax combined with decitabine or azacitidine in treatment-naive, elderly patients with acute myeloid leukemia. Blood 2019;133:7–17. 10.1182/blood-2018-08-868752. PMC6318429 30361262

[38] Moujalled DM , Brown FC, Chua CC, Dengler MA, Pomilio G, Anstee NS, . Acquired mutations in BAX confer resistance to BH3-mimetic therapy in acute myeloid leukemia. Blood 2023;141:634–44. 10.1182/blood.2022016090. PMC10651776 36219880

[39] Zang W , Koike Y, Nishimura K, Tanaka A, Yamazaki H, Yamasaki T, . BRD4 and MYB inhibition overcomes venetoclax resistance in EVI1-rearranged acute myeloid leukemia. Sci Rep 2025;15:37099. 10.1038/s41598-025-21034-1. PMC12549952 41131284

[40] Kawano G , Ikeda R, Ishihara D, Shima T, Sakoda T, Yamamoto S, . Guanine nucleotides drive ribosome biogenesis and glycolytic reprogramming in acute myeloid leukemia stem cells. Blood 2026;147:768–82. 10.1182/blood.2025030209. PMC12917303 41191529

[41] Morita M , Kudo K, Shima H, Tanuma N. Dietary intervention as a therapeutic for cancer. Cancer Sci 2021;112:498–504. 10.1111/cas.14777. PMC7893991 33340176

[42] Del Nagro CJ , Xiao Y, Rangell L, Reichelt M, O’Brien T. Depletion of the central metabolite NAD leads to oncosis-mediated cell death. J Biol Chem 2014;289:35182–92, 10.1074/jbc.M114.580159. PMC4271207 25355314

[43] Leist M , Single B, Castoldi AF, Kühnle S, Nicotera P. Intracellular adenosine triphosphate (ATP) concentration: a switch in the decision between apoptosis and necrosis. J Exp Med 1997;185:1481–6. 10.1084/jem.185.8.1481. PMC2196283 9126928

[44] Bieganowski P , Brenner C. Discoveries of nicotinamide riboside as a nutrient and conserved NRK genes establish a Preiss-Handler independent route to NAD^+^ in fungi and humans. Cell 2004;117:495–502. 10.1016/s0092-8674(04)00416-7. 15137942

[45] Fletcher RS , Ratajczak J, Doig CL, Oakey LA, Callingham R, Da Silva Xavier G, . Nicotinamide riboside kinases display redundancy in mediating nicotinamide mononucleotide and nicotinamide riboside metabolism in skeletal muscle cells. Mol Metab 2017;6:819–32. 10.1016/j.molmet.2017.05.011. PMC5518663 28752046

[46] Ratajczak J , Joffraud M, Trammell SAJ, Ras R, Canela N, Boutant M, . NRK1 controls nicotinamide mononucleotide and nicotinamide riboside metabolism in mammalian cells. Nat Commun 2016;7:13103. 10.1038/ncomms13103. PMC5476803 27725675

[47] Somers K , Evans K, Cheung L, Karsa M, Pritchard T, Kosciolek A, . Effective targeting of NAMPT in patient-derived xenograft models of high-risk pediatric acute lymphoblastic leukemia. Leukemia 2020;34:1524–39. 10.1038/s41375-019-0683-6. PMC9110273 31848452

[48] Nahimana A , Attinger A, Aubry D, Greaney P, Ireson C, Thougaard AV, . The NAD biosynthesis inhibitor APO866 has potent antitumor activity against hematologic malignancies. Blood 2009;113:3276–86. 10.1182/blood-2008-08-173369. 19196867

[49] Olesen UH , Thougaard AV, Jensen PB, Sehested M. A preclinical study on the rescue of normal tissue by nicotinic acid in high-dose treatment with APO866, a specific nicotinamide phosphoribosyltransferase inhibitor. Mol Cancer Ther 2010;9:1609–17. 10.1158/1535-7163.MCT-09-1130. 20515945

[50] Razak AR , Mahipal A, Diamond JR, Ribas A, Berlin JD, Azmi AS, . First-in-human phase I study of KPT-9274, a first-in-class dual inhibitor of PAK4 and NAMPT, in patients with advanced solid malignancies. Target Oncol 2026;21:187–98. 10.1007/s11523-026-01206-3. PMC13031219 41824279

[51] Wei Y , Xiang H, Zhang W. Review of various NAMPT inhibitors for the treatment of cancer. Front Pharmacol 2022;13:970553. 10.3389/fphar.2022.970553. PMC9490061 36160449

