# Supplementary material for: Nicotinic Acid Restriction Potentiates NAMPT Inhibition in Acute Myeloid Leukemia Xenograft Models
Source: Cancer Res Commun. 2026 Oct 28;6(10):2529–40. doi: 10.1158/2767-9764.CRC-26-0274 (PMC13715585; doi:10.1158/2767-9764.CRC-26-0274)
Supplement: Supplementary file 1 [file crc-26-0274_supplementary_figure_s1_suppsf1.pdf]

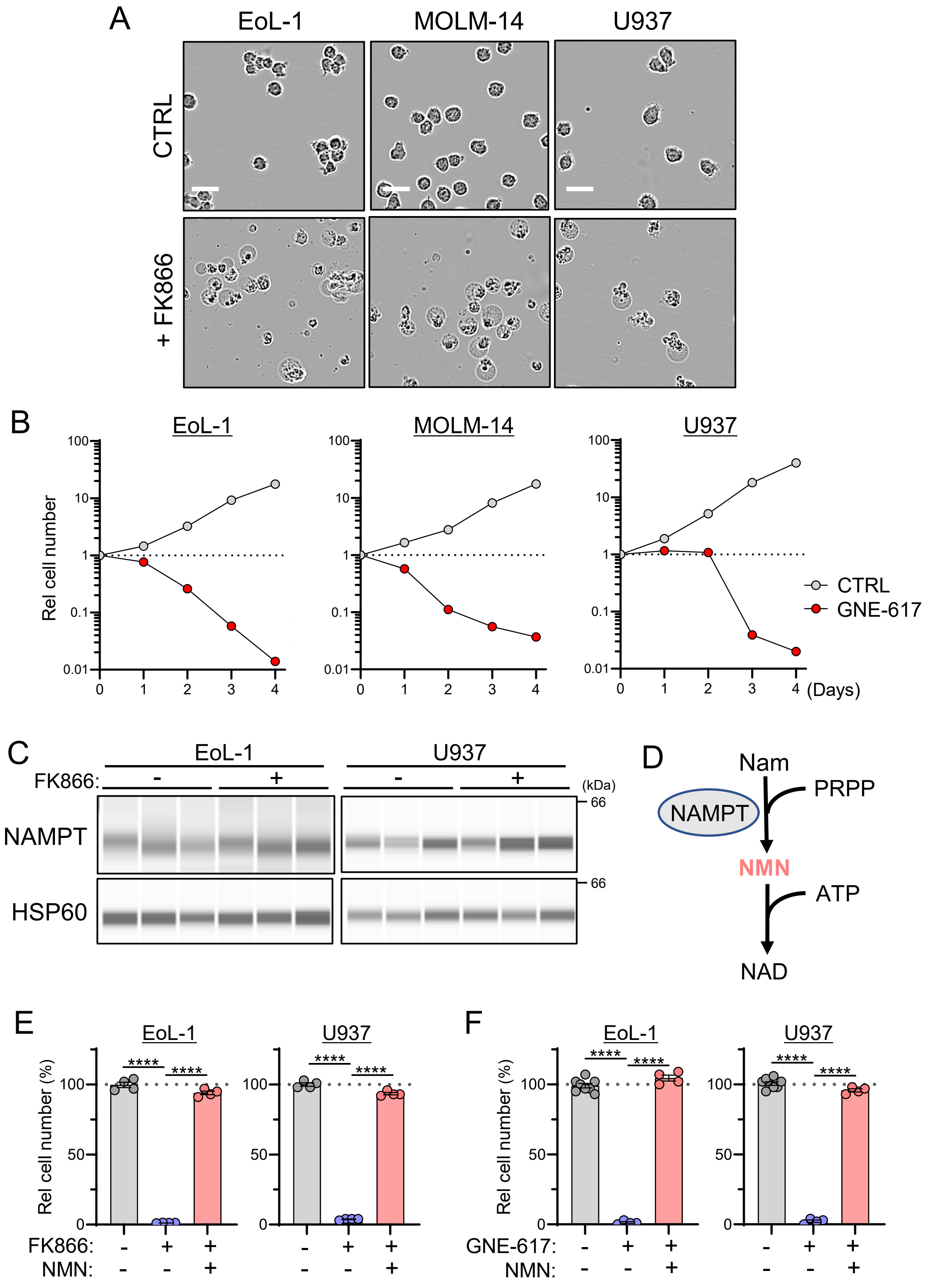

Supplementary Figure S1. Effects of NAMPTi on AML cell proliferation, related to Fig. 1.

(A) Representative phase contrast images of AML cells cultured 3 days with or without FK866. Scale bars, 25 µm.

(B) Effect of GNE-617 on AML cell proliferation. EoL-1, MOLM-14, and U937 cells were cultured with or without 10 nM GNE-617, and numbers of viable cells were determined over 4 days. Data are representative of two independent experiments and presented as the mean of 4 measurement replicates.

(C) Effect of FK866 on NAMPT protein levels. EoL-1 and U937 cells were treated with or without 20 nM FK866 for 24 h. NAMPT protein levels were analyzed by immunoassay. HSP60 served as a loading control. Each lane represents an independent biological replicate; n = 3.

(D) Schematic representation of the NAD salvage pathway. Nam, nicotinamide; PRPP, phosphoribosyl diphosphate; NMN, nicotinamide mononucleotide.

(E, F) Effect of NMN on NAMPTi-induced cytotoxicity. EoL-1 and U937 cells were treated 4 days with 20 nM FK866 (E) or 10 nM GNE-617 (F), in the presence or absence of 500 μM NMN, and then the number of viable cells was determined. Results are shown relative to untreated controls, which were defined as 100%. Data are presented as the mean of biological replicates plus SEM. $n = 8$ (untreated groups in F) or 4 (all others) biological replicates. ****$p < 0.0001$ as determined by one-way ANOVA with a post hoc test.
