# Supplementary material for: Nicotinic Acid Restriction Potentiates NAMPT Inhibition in Acute Myeloid Leukemia Xenograft Models
Source: Cancer Res Commun. 2026 Oct 28;6(10):2529–40. doi: 10.1158/2767-9764.CRC-26-0274 (PMC13715585; doi:10.1158/2767-9764.CRC-26-0274)
Supplement: Supplementary file 2 [file crc-26-0274_supplementary_figure_s2_suppsf2.pdf]

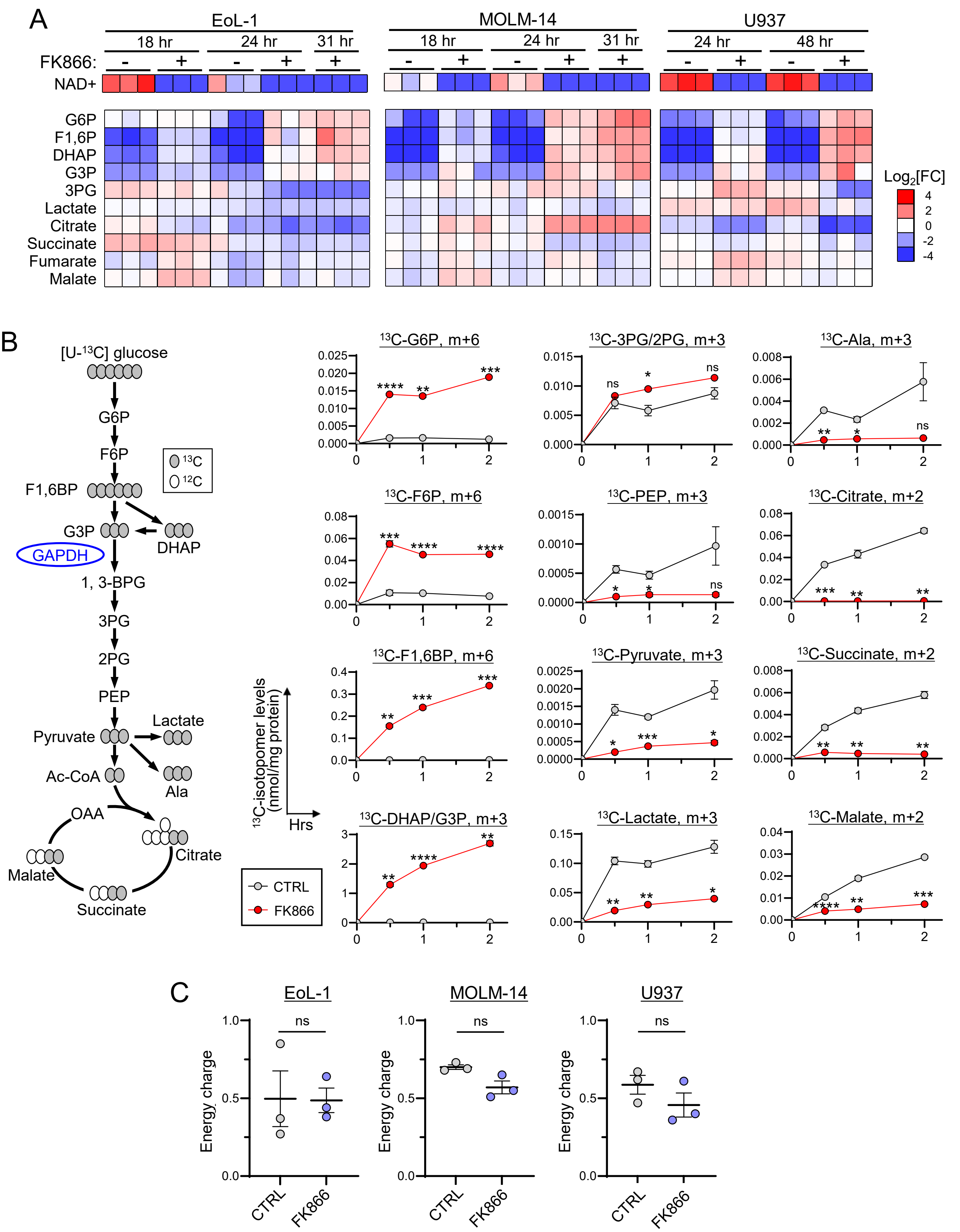

Supplementary Figure S2. Effects of FK866 on AML cell metabolism, related to Fig. 1.

(A) Relative levels of NAD^+, glycolytic metabolites, and TCA cycle intermediates in control and FK866-treated cells. Cells were treated with 20 nM FK866 for indicated times, or left untreated. Metabolite levels were measured by CE-MS. For each metabolite, data are presented as $\log_2$ fold-changes relative to the mean level across all samples. Each lane represents an independent biological replicate; n = 3.

(B) Time course analysis using [U-^{13}C] glucose as a tracer in EoL-1 cells. Cells were treated with or without 20 nM FK866 for 24 h, then labeled with [U-^{13}C] glucose up to an additional 2 h. Levels of indicated ^{13}C-labeled isotopomers are shown. n = 3 biological replicates. G6P, glucose-6-phosphate; F6P, fructose 6-phosphate; F1,6BP, fructose 1,6-bisphosphate; G3P, glyceraldehyde 3-phosphate; DHAP, dihydroxyacetone phosphate; 1,3-BPG, 1,3-bisphosphoglycerate; 3-PG, 3-phosphoglyceric acid; PEP, phosphoenolpyruvate; OAA, oxalacetic acid.

(C) Adenylate energy charge following FK866 treatment. Cells were treated with or without FK866 for 31 h (EoL-1 and MOLM-14) or 48 h (U937). Adenylate energy charge was calculated as described in Materials and Methods. n = 3 biological replicates.

In B and C, data are presented as the mean of biological replicates plus SEM. *p < 0.05, **p < 0.01, ***p < 0.001, ****p < 0.0001, as determined by a two-tailed t-test. ns., not significant.
