# Supplementary material for: Nicotinic Acid Restriction Potentiates NAMPT Inhibition in Acute Myeloid Leukemia Xenograft Models
Source: Cancer Res Commun. 2026 Oct 28;6(10):2529–40. doi: 10.1158/2767-9764.CRC-26-0274 (PMC13715585; doi:10.1158/2767-9764.CRC-26-0274)
Supplement: Supplementary file 3 [file crc-26-0274_supplementary_figure_s3_suppsf3.pdf]

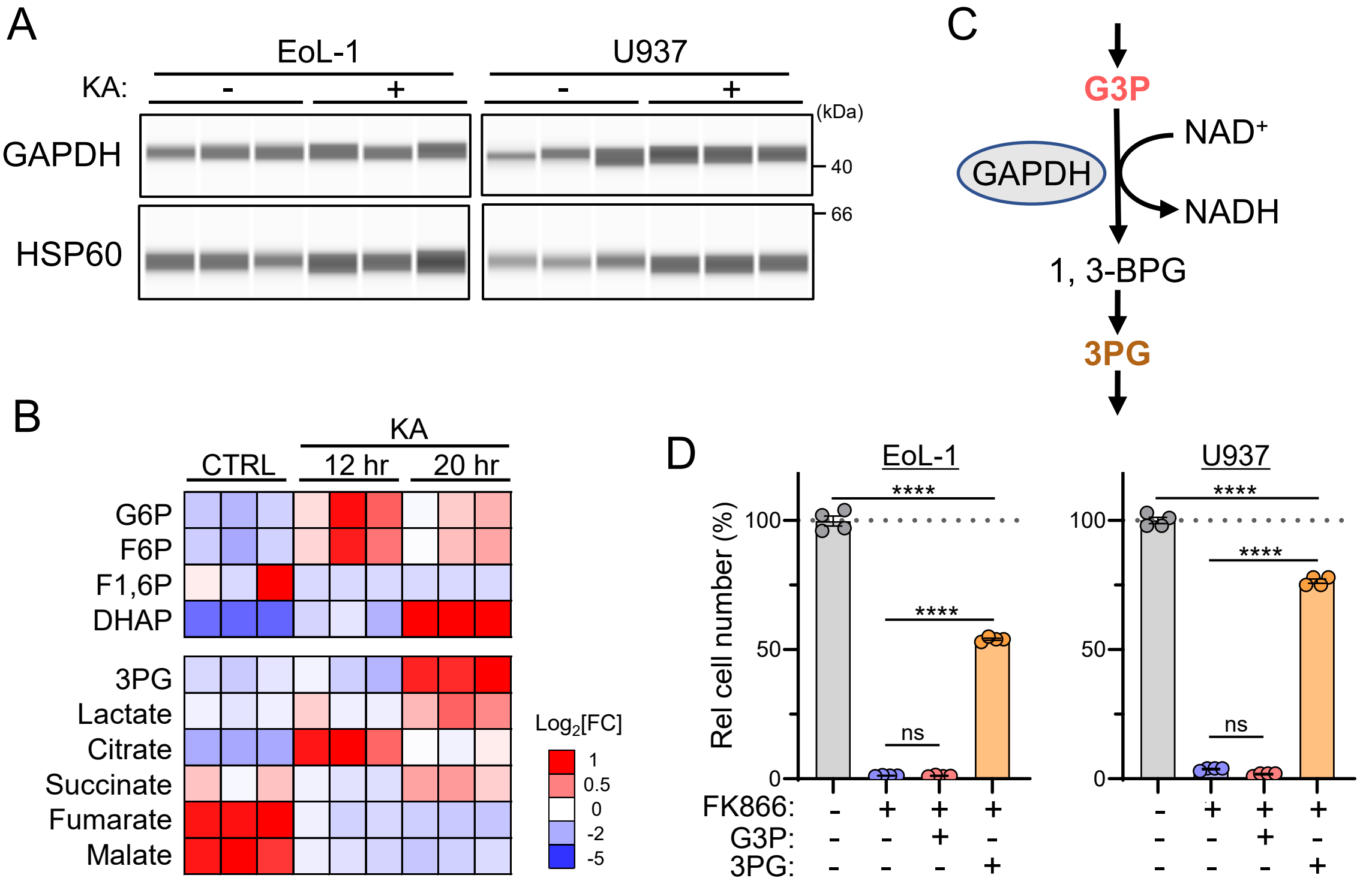

Supplementary Figure S3. Effects of KA and exogenous 3-PG, related to Fig. 1.

(A) Effect of KA on GAPDH protein levels. EoL-1 and U937 cells were treated with or without 5 µM KA for 24 h. GAPDH protein levels were analyzed by immunoassay. HSP60 served as a loading control. Each lane represents an independent biological replicate; n = 3.

(B) Effects of KA on levels of glycolytic and TCA metabolites. Cells were treated with 5 µM KA for indicated times. Metabolite levels were measured by CE-MS. For each metabolite, data are presented as $\log_2$ (fold-changes) relative to the mean level across all samples. Each column represents individual sample. n = 3 biological replicates.

(C) Schematic representation of the GAPDH-catalyzed step in glycolysis. G3P, glyceraldehyde 3-phosphate; 1,3-BPG, 1,3-bisphosphoglycerate; 3-PG, 3-phosphoglyceric acid.

(D) Effects of G3P and 3-PG on FK866-induced cytotoxicity. EoL-1 and U937 cells were treated for 4 days with 20 nM FK866, in the presence or absence of 1 mM G3P or 1 mM 3-PG, and then the number of viable cells was determined. Results are shown relative to untreated controls, which were defined as 100%. Data are presented as the mean of biological replicates plus SEM. n = 4 biological replicates. ****$p < 0.0001$, as determined by one-way ANOVA with a post hoc test. ns., not significant.
