# Supplementary material for: Nicotinic Acid Restriction Potentiates NAMPT Inhibition in Acute Myeloid Leukemia Xenograft Models
Source: Cancer Res Commun. 2026 Oct 28;6(10):2529–40. doi: 10.1158/2767-9764.CRC-26-0274 (PMC13715585; doi:10.1158/2767-9764.CRC-26-0274)
Supplement: Supplementary file 4 [file crc-26-0274_supplementary_figure_s4_suppsf4.pdf]

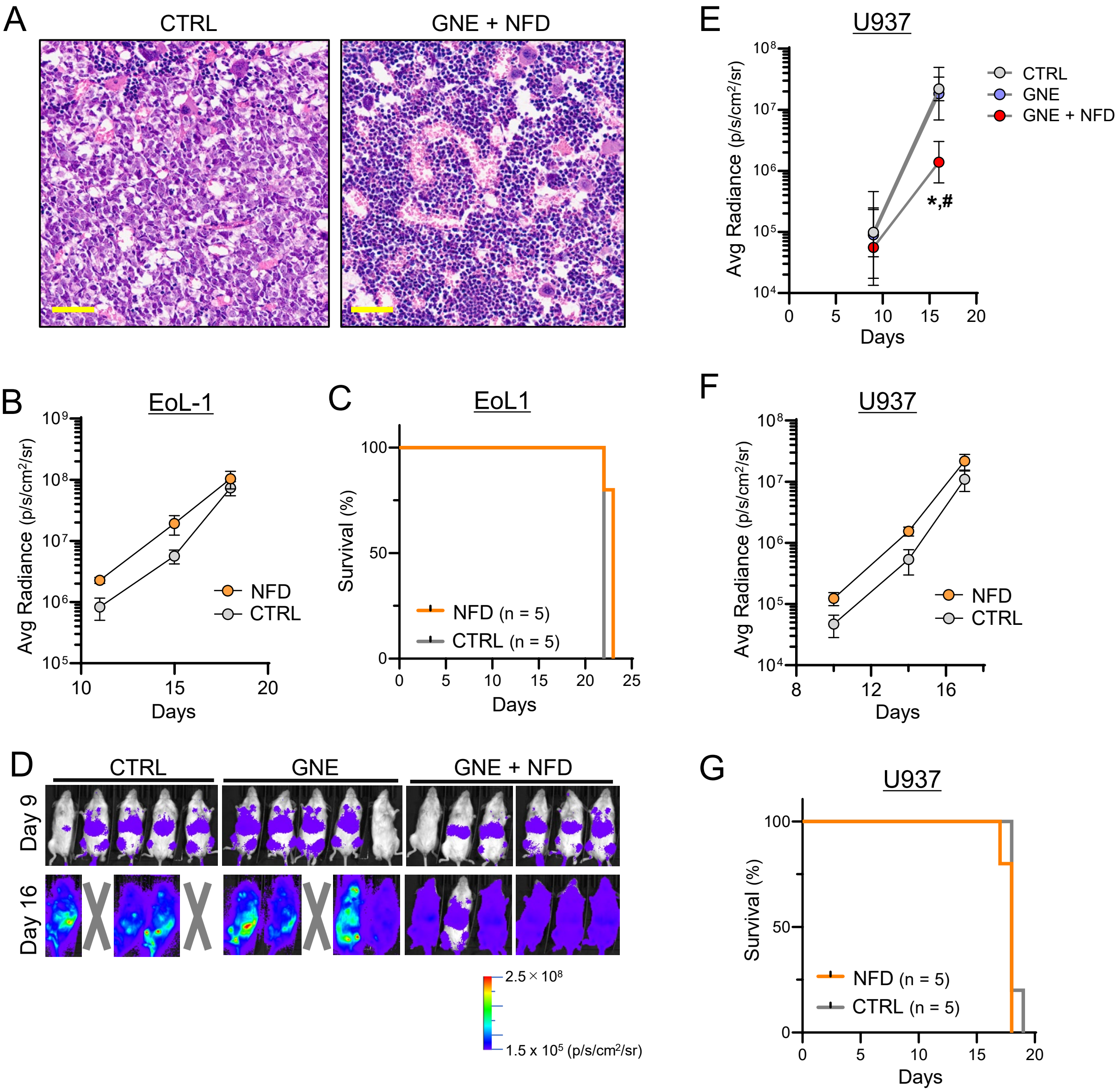

Supplementary Figure S4. Effects of NAMPTi and NFD on leukemic burden, related to Fig. 3.

(A) Histological analysis of femoral bone marrow. EoL-1/Luc-transplanted mice were treated with vehicle or GNE-617 plus NFD. Femurs were collected on day 15 after transplantation, sectioned and stained with H&E. Images are representative of 3 mice per group. Scale bars, 50 µm.

(B) Effect of NFD alone on leukemic burden in EoL-1/luc xenograft mice. Mice were fed a normal diet (CTRL) or NFD, and leukemic burden was monitored by bioluminescent imaging on indicated days. n = 5 mice per groups.

(C) Survival of the mice shown in B.

(D) Bioluminescence imaging of U937-luc-transplanted mice on indicated days. Mice were treated as shown in Fig. 3B. n = 5 (CTRL, GNE) or 6 mice (GNE + NFD).

(E) Quantification of leukemic burden on days 9 and 16, based on bioluminescent imaging in D.

(F) Effect of NFD alone on leukemic burden in U937/luc xenograft mice. Mice were treated as in B. n = 5 mice per groups.

(G) Survival of mice shown in F.

In B, E and F, data are presented as the mean of biological replicates plus SEM. *$p < 0.05$ and #$p < 0.05$ relative to CTRL and GNE, respectively, as determined by a two-tailed t-test.
