# Supplementary material for: Nicotinic Acid Restriction Potentiates NAMPT Inhibition in Acute Myeloid Leukemia Xenograft Models
Source: Cancer Res Commun. 2026 Oct 28;6(10):2529–40. doi: 10.1158/2767-9764.CRC-26-0274 (PMC13715585; doi:10.1158/2767-9764.CRC-26-0274)
Supplement: Supplementary file 5 [file crc-26-0274_supplementary_figure_s5_suppsf5.pdf]

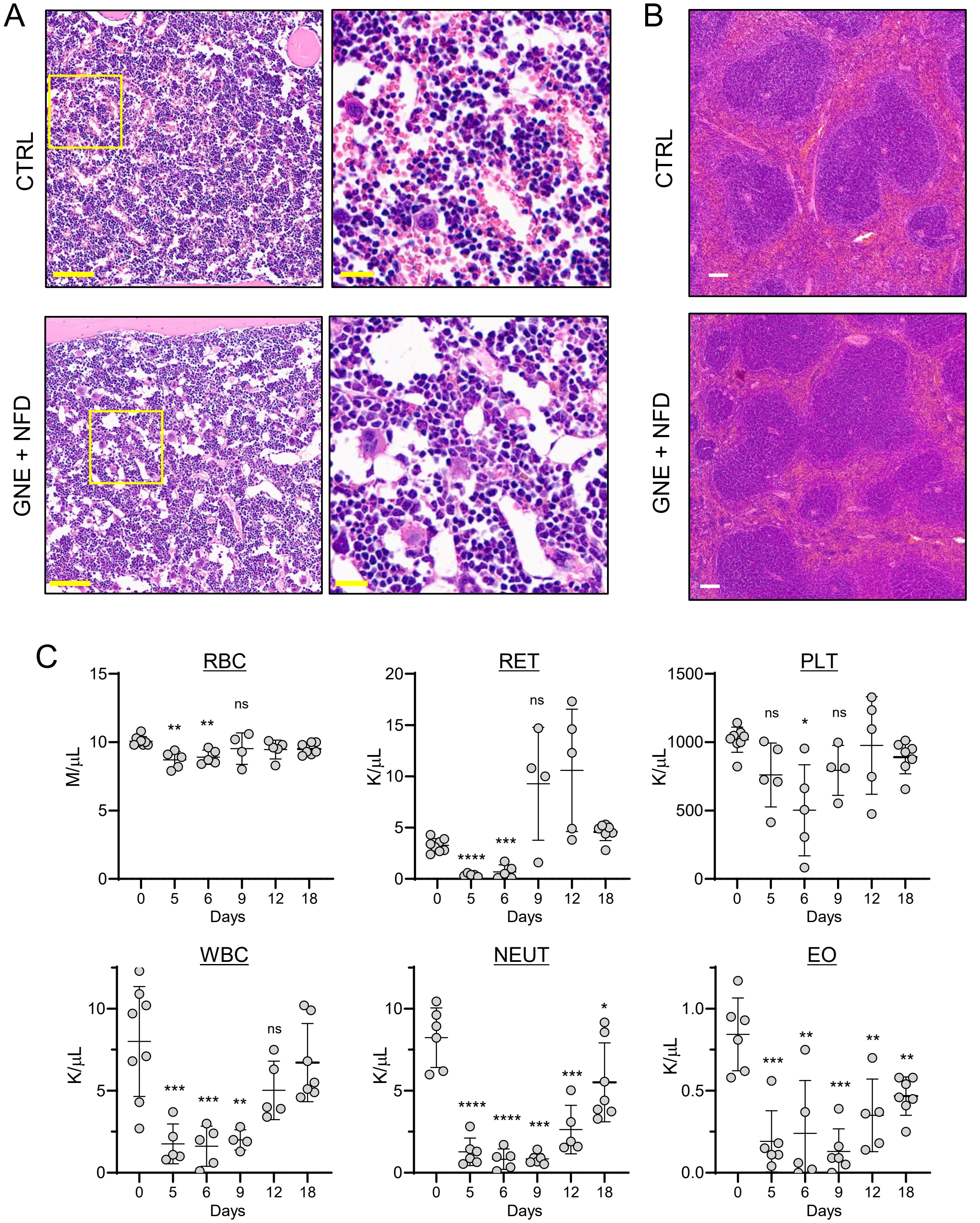

Supplementary Figure S5. Effects of NAMPTi and NFD on hematopoiesis, related to Fig. 4.

(A) Histological analysis of femoral bone marrow. Femurs were collected on day 5 from mice in CTRL and GNE + NFD groups, and bone marrow sections were stained with H&E. Boxed regions in the low-magnification images (left) are shown at higher magnification at right. Images are representative of four mice per group. Scale bars, 100 µm (left) and 20 µm (right).

(B) Histological analysis of spleen. Spleens were collected on day 5 from mice in CTRL and GNE + NFD groups, and tissue sections were stained with H&E. Images are representative of four mice per group. Scale bars, 100 µm.

(C) Time course showing absolute peripheral blood cell counts in mice treated with GNE-617 plus NFD. Counts of RBC, RET, WBC, PLT, NEUT, and EO are shown. Each symbol represents an individual mouse. Data are presented as the mean ± SEM; n = 5-8 mice per time point. *$p < 0.05$, **$p < 0.01$, ***$p < 0.001$ and ****$p < 0.0001$ relative to CTRL as determined by a two-tailed t-test.
